# Decidual Macrophage Functional Polarization during Abnormal Pregnancy due to *Toxoplasma gondii*: Role for LILRB4

**DOI:** 10.3389/fimmu.2017.01013

**Published:** 2017-08-24

**Authors:** Zhidan Li, Mingdong Zhao, Teng Li, Jing Zheng, Xianbing Liu, Yuzhu Jiang, Haixia Zhang, Xuemei Hu

**Affiliations:** ^1^Department of Immunology, Medicine & Pharmacy Research Center, Binzhou Medical University, Yantai, China; ^2^Department of Radiology, Binzhou Affiliated Hospital of Binzhou Medical University, Binzhou, China; ^3^Department of Gynecology and Obstetrics, Yantai Traditional Chinese Medicine Hospital, Yantai, China

**Keywords:** inhibitory receptor, LILRB4, *Toxoplasma gondii*, abnormal pregnancy outcomes, decidual macrophage

## Abstract

During gestation, *Toxoplasma gondii* infection produces a series of complications including stillbirths, abortions, and congenital malformations. The inhibitory receptor, LILRB4, which is mainly expressed by professional antigen-presenting cells (especially macrophages and dendritic cells) may play an important immune-regulatory role at the maternal–fetal interface. To assess the role of LILRB4 during *T. gondii* infection, LILRB4^−/−^ and *T. gondii* infected pregnant mouse models were established. Further, human primary-decidual macrophages were treated with anti-LILRB4 neutralizing antibody and then infected with *T. gondii*. These *in vivo* and *in vitro* models were used to explore the role of LILRB4 in *T. gondii-*mediated abnormal pregnancy outcomes. The results showed that abnormal pregnancy outcomes were more prevalent in LILRB4^−/−^ infected pregnant mice than in wild-type infected pregnant mice. In subsequent experiments, expression levels of LILRB4, M1, and M2 membrane-functional molecules, arginine metabolic enzymes, and related cytokines were assessed in uninfected, infected, LILRB4-neutralized infected, and LILRB4^−/−^ infected models. The results demonstrated *T. gondii* infection to downregulate LILRB4 on decidual macrophages, which strengthened M1 activation functions and weakened M2 tolerance functions by changing M1 and M2 membrane molecule expression, synthesis of arginine metabolic enzymes, and cytokine secretion profiles. These changes contributed to abnormal pregnancy outcomes. The results of this study provide not only a deeper understanding of the immune mechanisms operational during abnormal pregnancy, induced by *T. gondii* infection, but also identify potential avenues for therapeutic and preventive treatment of congenital toxoplasmosis.

## Introduction

*Toxoplasma* is one of the TORCHES (toxoplasmosis, rubella, cytomegalovirus, herpes simplex, syphilis). It is an obligate, intracellular, protozoan parasite that is widespread in nature and a frequent human pathogen (1). *T. gondii* infection is initially asymptomatic, but immune alterations accompany infection, and these result in adverse outcomes. In particular, infections during early pregnancy can result in premature births, miscarriages, anencephaly, and other adverse pregnancy outcomes (2, 3). The mechanism by which infection with *T. gondii* contributes to these adverse pregnancy outcomes is unknown.

Like a successful allograft, a specialized immune microenvironment is crucial to the maintenance of normal pregnancy (4). The microenvironment contains decidual immunological cell populations including macrophages, natural killer cells (NK), T regulatory cells (Treg), and dendritic cells (DC), which secrete immune-suppressive cytokines such as interleukin 10 (IL-10) and transforming growth factor beta (5, 6). Macrophages comprise 20–25% of these decidual immunological cell populations. These macrophages maintain some relatively stable functional molecules and cytokine expression profile, permitting maternal–fetal tolerance throughout gestation (7, 8). Macrophages are generally categorized as classically activated M1 type or alternatively activated M2 type macrophages (9, 10). In normal pregnancy, the M2 type is the major decidual macrophage population and these cells express the surface markers CD163, CD209, and 206. These macrophages secrete IL-10, which promotes immune tolerance, and also synthesize the arginine metabolic enzyme type-I arginase (Arg-I), which is involved in tissue remolding (11). In contrast, M1 type decidual macrophages have high levels of CD80 and CD86 expression. These macrophages secrete tumor necrosis factor alpha (TNF-α) and synthesize inducible nitric oxide synthase (iNOS), which promote a pro-inflammatory response (12). In mice, M2 decidual macrophages are characterized by expression of CD206^high^, IL-10^high^, and Arg-I^high^, whereas M1 decidual macrophages are characterized by expression of CD80^high^, CD86^high^, TNF-α^high^, and iNOS^high^ (12).

Human leukocyte immunoglobulin-like receptor subfamily B member 4 (LILRB4), or gp49B (in mice), is highly expressed on macrophages and is a member of the immunoglobulin-inhibitory receptor superfamily. LILRB4 contains putative immune receptor, tyrosine-based, inhibitory motifs, which recruit inhibitory phosphatases, transducing negative signals within cells (13, 14). High expression levels of LILRB4 are crucial for immune tolerance and immune regulation during normal pregnancy (15, 16). Acting as an endogenous negative regulator of macrophage activation, LILRB4 ligation reportedly inhibits TNF-α production, which modulates decidual macrophage function in mice (17).

Studies have reported that LILRB4^−/−^ mice were born at expected ratios, were healthy and fertile, and displayed normal long-term survival rates (18). It is unknown whether decidual macrophage LILRB4 is involved in abnormal pregnancy outcomes due to *T. gondii*. In this study, LILRB4^−/−^ pregnant mice were infected with *T. gondii*, and the role of macrophage LILRB4 in abnormal pregnancy was assessed. Further, human decidual macrophages were infected with *T. gondii* in order to evaluate the potential involvement of LILRB4 in adverse human pregnancy outcomes.

## Materials and Methods

### Ethics Statement

Sample collection procedures for this study were approved by the Binzhou Medical University Ethics Committee (Shandong, China). All subjects provided written informed consent for the collection of samples and subsequent analysis. The Ethics Committees approved this consent procedure. This study was carried out in strict accordance with the recommendations in the Guide for the Care and Use of Laboratory Animals of Binzhou Medical University. The protocol was approved by the Committee on the Ethics of Animal Experiments of Binzhou Medical University. All procedures were performed under sodium pentobarbital anesthesia, and all efforts were made to minimize suffering of the animals.

### Animal Models

C57BL/6 wild-type mice were purchased from Beijing Vital River Laboratory Animal Technology Co., Ltd. (Beijing, China). LILRB4-deficient (LILRB4^−/−^) C57BL/6 mice were obtained from Riken BioResource Center (Tsukuba, Japan). Subsequently, 6- to 8-week-old female mice were housed five per cage, and 8- to 10-week-old male mice were housed one per cage. All mice were maintained in the specific pathogen-free animal house of Binzhou Medical University at 22–26°C with 50–60% humidity and a 12 h light/12 h dark cycle, with abundant sterilized water and food (Jiangsu Biological Engineering Co., Ltd., China). One day after cohabitation with males (at a ratio of 2 females:1 male), female mice with vaginal plugs [gestational day (gd) 0] were segregated and randomized into uninfected, infected with *T. gondii*, and LILRB4^−/−^ infected with *T. gondii* groups.

### Genotyping of LILRB4^−/−^ Mice

Genomic DNA was extracted from mouse tails using a tissue DNA extraction kits (Generay, China). Polymerase chain reaction (PCR) was used to synthesize cDNA. After initial denaturation (3 min at 95°C), PCR was performed with 35 amplification cycles of denaturation for 30 s at 95°C, annealing for 30 s at 55°C, and extension for 60 s at 72°C, followed by a final extension for 5 min at 72°C. PCR products were separated by electrophoresis in 2% agarose gels, and sizes were estimated using Trans DNA marker I (100–700 bp; Transgene, France). Gels were stained with GelStain (10,000×; Transgene, France) to visualize DNA. Primers for PCR amplification were LILRB4 P1, 5′-ACCGGTGGATGTGGAATGTGTG-3′; LILRB4 P2, 5′-GTCCTGGGTTCCAGAATAAGAC-3′; and LILRB4 P3, 5′-TCTGCTCTTAGGAAATTACAGAA-3′.

The expected PCR product sizes were 260 bp (mutant), 371 and 260 bp (heterozygote), and 371 bp (wild-type). Homozygous LILRB4^−/−^ mice were continually bred for the duration of the study.

### Preparation of *T. gondii* RH Tachyzoites

*Toxoplasma gondii* tachyzoites were maintained in HEp-2 cells in minimum essential media (MEM) (Hyclone, USA), 5% fetal bovine serum (FBS; Gibco, USA), and 100 IU/ml penicillin/streptomycin, which were purchased from Sigma-Aldrich (USA). After culture, tachyzoites were centrifuged at 1,500 rpm (433 × *g*) for 10 min, and purified tachyzoites were resuspended in MEM and counted using a Neubauer chamber.

### Infection Models and Pregnancy Outcomes Assessment

Pregnant mice (either LILRB4^−/−^ infected or wide-type infected groups of mice) were inoculated intraperitoneally (i.p.) with 400 tachyzoites in 200 µl of sterile phosphate buffer (PBS) on gd 8. Uninfected groups of mice were inoculated with 200 µl of sterile PBS. All mice were sacrificed at 6 days post-infection (dpi), uteri were removed, and the total number of implantation and resorption sites counted. Resorption sites were identified by their small size and the necrotic and hemorrhagic appearance of the embryos. Placenta were compared with those of uninfected mice. Abortion rates were calculated as the ratio of resorption sites to total implantation sites (resorptions plus normal implantation sites).

### Cell Preparation

Embryos and placenta were dissected and removed from mice at gd 14. Dispersed cells were prepared from small pieces of placenta and uterine tissue using a GentleMACS dissociator (Miltenyi, Germany). Single cell suspensions were then obtained by filtration through 48 µm sterile nets. After Ficoll density gradient centrifugation in mouse lymphocyte separation medium (TBD Science, China), mononuclear cells were collected from the white film layer and assessed by flow cytometry.

### Human Clinical Sample Collection

Decidual tissues were taken from women without evidence of clinical genital infections during pregnancy. All voluntary abortions occurred in the Department of Obstetrics and Gynecology, Yantai Hospital of Traditional Chinese Medicine and Yantai Affiliated Hospital of Binzhou Medical University between 8 and 10 weeks after conception. Sample collection protocols were approved by the ethics Committee of Binzhou Medical University. Tissues were rinsed with sterile saline solution 5–8 times, and decidual tissues were picked and cultured in Dulbecco’s modified eagle medium/high glucose medium (Hyclone, USA) supplemented with 100 IU/ml penicillin and 100 IU/ml streptomycin (Sigma-Aldrich, USA).

### Isolation of Human Decidual Macrophages

Decidual tissues were immediately washed 5–6 times in Roswell Park Memorial Institute (RPMI) medium, cut into pieces, and then digested in 0.1% collagenase type IV (Sigma-Aldrich, USA) solution containing 25 IU/ml DNase-I (Sigma-Aldrich, USA) at 37°C for 30 min. Single cell suspensions were obtained using a GentleMACS dissociator (Miltenyi Biotech, Germany) and were filtered through 48 µm nylon mesh filters. Density gradient centrifugation was then performed using human lymphocyte separation medium (TBD Science, China) at 2,000 rpm (771 × *g*) for 20 min at 20°C according to the manufacturer’s instructions for mononuclear cell collection. Decidual macrophages were then purified using a human CD14 positive selection kit (Stem Cell Science, USA) according to the manufacturer’s instructions with purity of >95% for all experiments. Human decidual macrophages were counted, and aliquots containing more than 1.5 × 10^6^ cells were allocated to uninfected, infected, and LILRB4-neutralized infected groups. Cells of the LILRB4-neutralized infected group were treated with neutralizing anti-LILRB4 antibody (10 µg/mL) for 1 h prior to *T. gondii* infection, which was performed at a ratio of 2:1, *T. gondii* to decidual macrophages. Samples containing approximately 5.0 × 10^5^ decidual macrophages were cultured in RPMI medium supplemented with 10% FBS (Gibco, USA), and 100 IU/ml penicillin and 100 IU/ml streptomycin (Sigma-Aldrich, USA) for 24 h at 37°C in a humidified 5% CO_2_ incubator.

### Flow Cytometry

The following fluorochrome-conjugated, mouse-specific monoclonal antibodies (mAbs) were used for assessment: Pe-cy7-conjugated anti-F4/80 (marker of mouse-decidual macrophage), PE-conjugated anti-LILRB4, FITC-conjugated anti-CD206, FITC-conjugated anti-CD86, FITC-conjugated anti-CD80, APC-conjugated anti-TNF-α (all from BioLegend, USA), APC-conjugated anti-IL-10 (Becton Dickinson, BD, USA), APC-conjugated anti-iNOS (eBioscience, USA), and APC-conjugated anti-Arg-I (RD, USA). Isolated mononuclear cells were incubated with anti-F4/80, anti-LILRB4, and anti-CD206; or anti-F4/80, anti-LILRB4, and anti-CD80; or anti-F4/80, anti-LILRB4, and anti-CD86 mAbs at 4°C in the dark for 30 min and were then washed twice. To stain intracellular Arg-I and iNOS, cells were first incubated with anti-F4/80 and anti-LILRB4 mAbs at 4°C in the dark for 30 min and then washed. Subsequently, cells were fixed and permeabilized in 1× Fix/Perm buffer (eBioscience, USA) for 30 min according to the manufacturer’s instructions. After washing twice, cells were incubated with anti-Arg-I and anti-iNOS mAbs at 4°C in the dark for 45 min and were then washed twice. For analysis of the intracellular cytokines IL-10 and TNF-α, cells were initially stimulated for 4 h with leukocyte activation cocktail, with BD GolgiPlug (BD, USA). Cells were then collected and incubated with anti-F4/80 and anti-LILRB4 mAbs at 4°C in the dark for 30 min and then washed. The cells were then fixed and permeabilized in 1× Fix/Perm buffer (eBioscience, USA) at 4°C for 30 min according to the manufacturer’s instructions. After washing twice, cells were incubated with anti-IL-10 or anti-TNF-α mAbs at 4°C in the dark for 45 min and washed.

Assays were performed with the following fluorochrome-conjugated, human-specific mAbs: Pe-cy7-conjugated anti-CD14, APC-conjugated anti-LILRB4, and PE-conjugated anti-TNF-α, all from eBioscience, USA. FITC-conjugated anti-CD206, FITC-conjugated anti-CD163, FITC-conjugated anti-CD209, PE-conjugated anti-CD80, PE-conjugated anti-CD86, and PE-conjugated anti-IL-10 were from BD, USA. Similarly, decidual macrophages were incubated with anti-CD14, anti-LILRB4, and anti-CD206; or anti-CD14, anti-LILRB4, and anti-209, or anti-CD14, anti-LILRB4, and anti-CD163, or anti-CD14, anti-LILRB4, and anti-CD80, or anti-CD14, anti-LILRB4, and anti-CD86 mAbs at 4°C in the dark for 30 min and were then washed once. Decidual macrophages were also collected and incubated with anti-CD14, anti-LILRB4, and anti-IL-10, or anti-TNF-α mAbs (as described above for intracellular cytokine staining). Analysis was performed with a FACScanto™ II instrument (Becton Dickinson, USA).

### Enzyme-Linked Immunosorbent Assays (ELISA)

Purified human decidual macrophages from uninfected, infected, and LILRB4-neutralized infected groups were cultured for 24 h after *T. gondii* infection, at a ratio of *T. gondii* to macrophage of 2:1. Cells were harvested from suspensions, and IL-10 and TNF-α levels were analyzed by ELISA according to the manufacturer’s protocols. Standard curves were generated using standards for each assay, and all measurements of absorbance were performed in triplicate at 450 nm. Concentrations were calculated according to standard curves and respective formulas.

### Western Blot

Purified human decidual macrophages were harvested after treatment and washed with PBS, then lysed for 40 min on ice in 100 µl of lysis buffer containing phenylmethanesulfonyl fluoride at 16:1 (Beyotime Biotech, China). Cell lysates were centrifuged at 12,000 × *g* for 20 min at 4°C, and supernatants were mixed with 5× sodium dodecyl sulfate-polyacrylamide (SDS-PAGE) loading buffer and boiled for 5 min. Aliquots containing 40 µg of protein were separated on 12% (for detection of Arg-I) or 8% (for detection of iNOS) SDS-PAGE gels. Proteins were then transferred onto polyvinylidene fluoride membranes (Millipore, USA) and were blocked with 7% non-fat dry milk in Tris-buffered saline containing 0.2% Tween-20 (TBS-T) at room temperature for at least 4 h. Membranes were then incubated with rabbit anti-human arginase-I and iNOS antibodies (1:1,000 dilution; Abcam, USA) at 4°C overnight. Membranes were then washed four times in 1× TBS-T for 10 min and incubated with horseradish peroxidase conjugated goat anti-rabbit IgG secondary antibody (1:2,000 dilution; Protech, USA) at room temperature for 1 h. After washing three times for 15 min, proteins on membranes were visualized using an enhanced chemiluminescence kit (ECL; F. Hoffmann-La Roche Ltd., Switzerland) at various exposure times. GAPDH was detected using rabbit anti-GAPDH polyclonal antibody (Protech, USA) as a loading control.

### Pathology Assessments

Mouse placentas and uteri were removed after treatments and were washed three times and fixed with 4% paraformaldehyde immediately. Tissues were then washed in running water and placed in a graded ethanol series of 30, 50, and 70% and were then paraffin embedded using standard methods. Paraffin sections were stained with hematoxylin and eosin dye (H&E; Shanghai Novland Co., Ltd., China) according to the manufacturer’s instructions. Images of paraffin embedded sections were recorded at 20× magnification and are presented with 5 µm scale bars.

### Immunofluorescence Imaging

Purified, human decidual macrophages from uninfected, infected, and LILRB4-neutralized infected groups were cultured for 24 h after *T. gondii* infection and then fixed in 4% paraformaldehyde for 15 min and blocked with goat serum for 1 h. Cells were then incubated overnight at 4°C with anti-LILRB4 (Santa Cruz, Germany) and anti-CD163 (BD, USA), or anti-LILRB4 and anti-CD86 (BD, USA) antibodies. DyLight 488-goat anti-rabbit IgG (Abbkine, USA) was used as a secondary antibody for anti-LILRB4 antibody, and DyLight 649-goat anti-mouse IgG (Abbkine, USA) was used as a secondary antibody for anti-CD163 and anti-CD86. Cells were incubated with appropriate concentrations of secondary antibodies at 37°C for 1 h and were subsequently stained with the nucleic acid stain 4′,6-diamidino-2-phenylindole for 15 min. Finally, cells were observed using a laser confocal microscope (Leica, Germany).

### Data Analysis and Statistics

Data are presented as means ± SD. Statistical analyses were performed using the GraphPad prism 5 statistics software package. Differences were identified using unpaired *t*-tests and were considered significant when two-tailed *p* values were less than 0.05 or very significant when two-tailed *p* values were less than 0.01.

## Results

### Animal Models and Abnormal Pregnancy Outcomes

**Figure 1:**
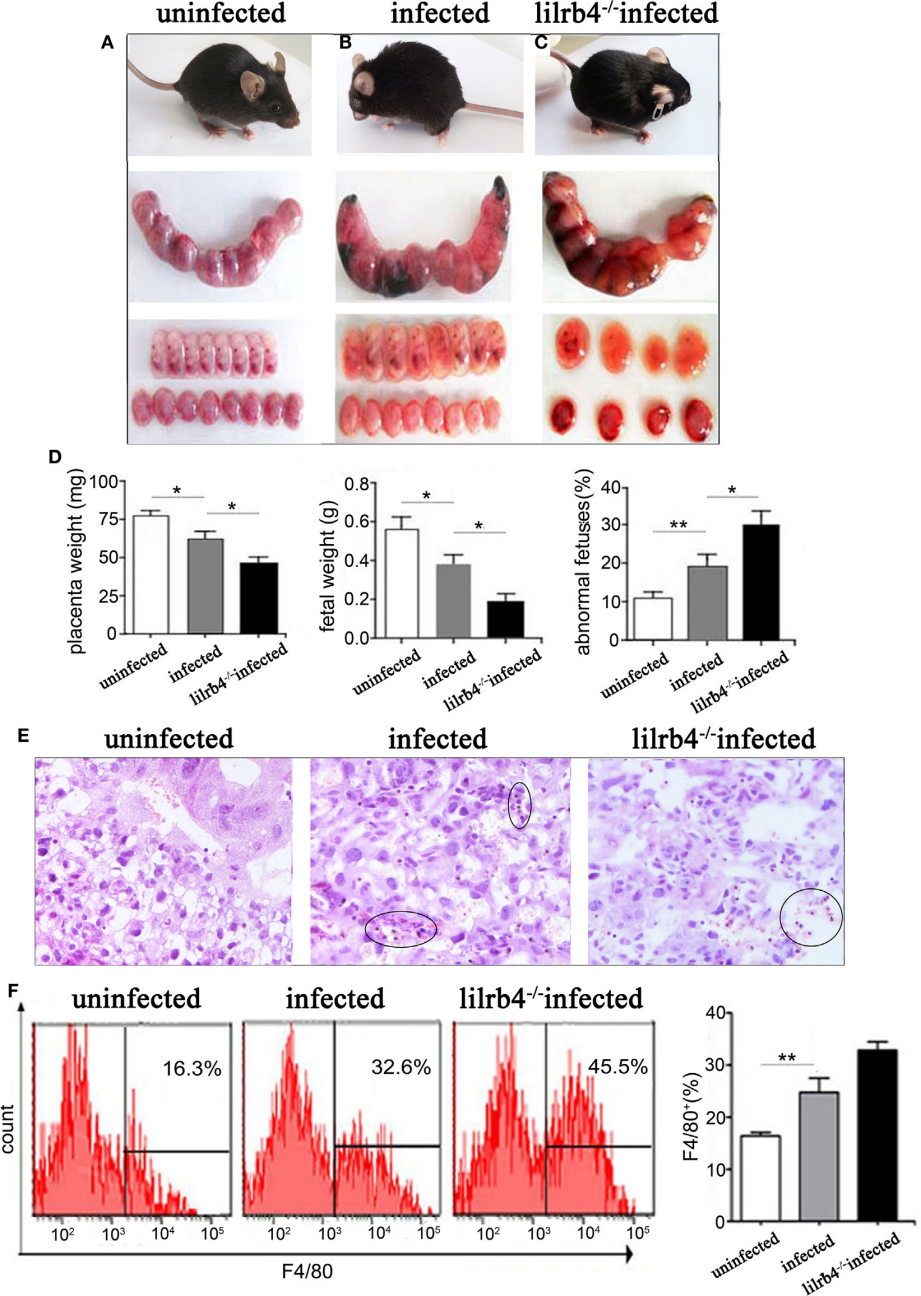
Effects of LILRB4 on pregnancy outcomes during *Toxoplasma gondii* infection in mice. **(A)** Uninfected mice were energetic and had well-developed fetuses and placentas. **(B)** Infected mice were lethargic, had smaller fetuses and placentas, and had poorer blood supply. **(C)** LILRB4^−/−^ infected mice were listless, and their fetuses and placentas were almost shapeless. **(D)** Placental and fetal weights and the percentage of abnormal fetuses are presented in uninfected, infected, and LILRB4^−/−^ infected mice. Abnormalities included stillbirths and resorption sites and were calculated as ratios of stillbirths and resorption sites to total number of implantation sites. **(E)** Hematoxylin and eosin (H&E) staining of representative placentas from uninfected, infected, and LILRB4^−/−^ infected mice, demonstrating hemorrhage and lymphocytic infiltration (black circle). **(F)** Percentages of F4/80^+^ decidual macrophages in uninfected, infected, and infected LILRB4^−/−^ mice were calculated by flow cytometric analysis. Representative data were derived from separate uninfected, infected, and LILRB4^−/−^ infected mice. Data are presented as means ± SD of 10 pregnant mice per group. Asterisks indicate significant differences for unpaired *t*-tests; *p < 0.05, **p < 0.01.

Infected pregnant mice were unkempt, had less mobility, erect fur, and placentas that were significantly inflamed with hyperemia. Absorbed fetuses and stillbirths were more prevalent in infected mice than in uninfected pregnant mice (Figures 1A,B). Infected mice had significantly lower placental and fetal weights as well as higher abnormal fetal ratios than uninfected mice (Figure 1D). Paraffin embedded sections of infected but not uninfected placentas showed damage as hemorrhage and lymphocyte infiltration, with spiral arteries (Figure 1E). In LILRB4^−/−^ infected mice, pregnancy outcomes were worse than outcomes observed in infected wild-type mice, which included more severe placental ischemia. Relative to wide-type infected mice, LILRB4^−/−^ infected fetuses were almost shapeless (Figures 1B,C), with reduced placental and fetal weights and abnormal fetal ratios (Figure 1D). Hemorrhage and lymphocyte infiltration were further exacerbated in the LILRB4^−/−^ infected mice compared with infected wide-type mice (Figure 1E). Moreover, the percentage of decidual macrophages in all infected groups of mice was significantly higher than in the uninfected mice but did not differ significantly from that of the LILRB4^−/−^ infected mice (Figure 1F).

### LILRB4 Expression by Decidual Macrophages Is Downregulated after *T. gondii* Infection

**Figure 2:**
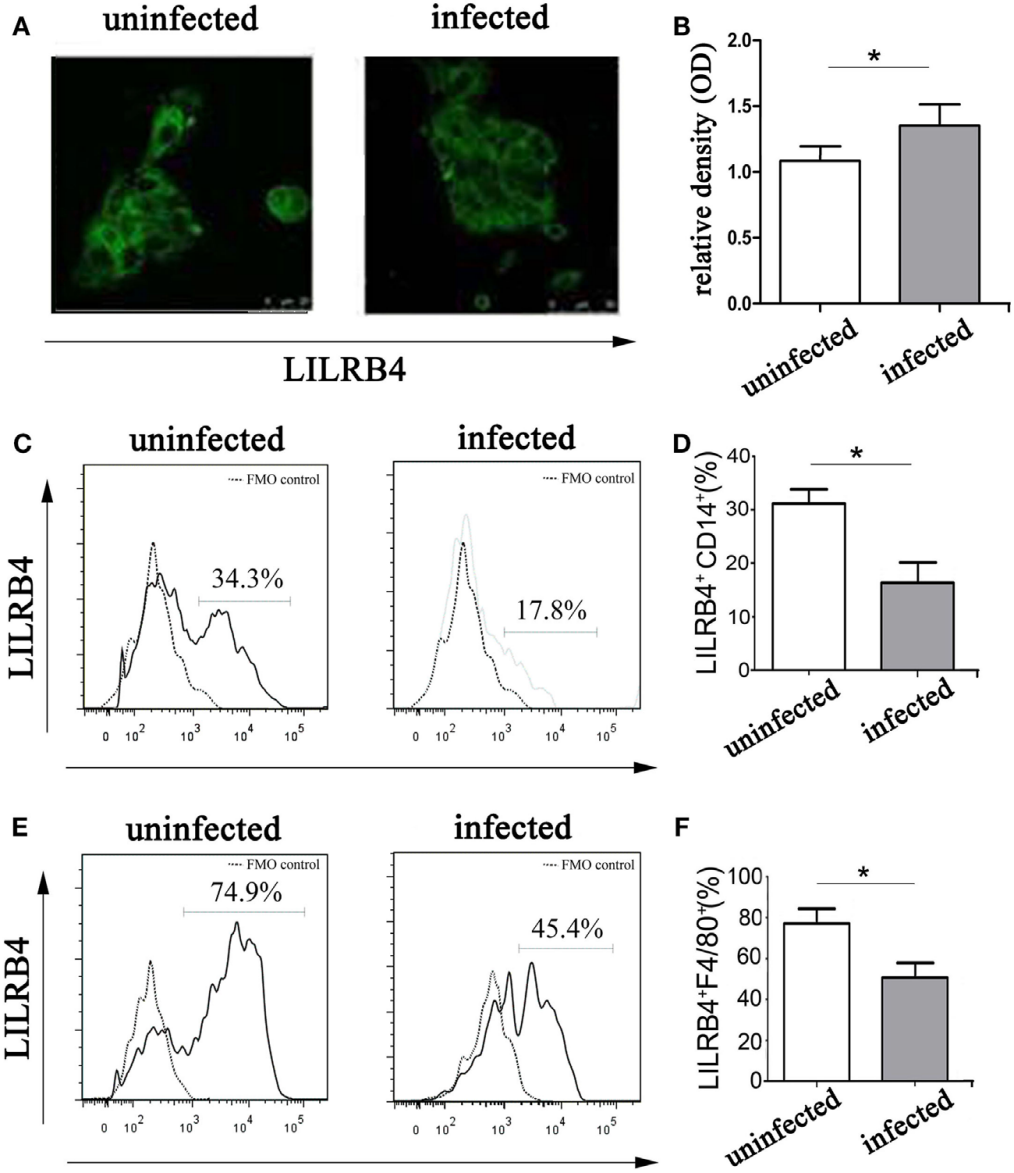
Expression of LILRB4 on decidual macrophages infected with *Toxoplasma gondii*. **(A)** LILRB4 expression levels in uninfected and infected human decidual macrophages were compared by immunofluorescence. **(B)** Histogram analysis of immunofluorescence for LILRB4 expression levels in uninfected and infected human decidual macrophages. **(C)** LILRB4 expression levels in uninfected and infected human decidual macrophages were compared by flow cytometry analyses. The flow cytometric FMO as the control. **(D)** Histogram analysis of flow cytometry for LILRB4 expression levels in uninfected and infected human decidual macrophages. **(E)** Flow cytometric analysis of LILRB4 expression changes in uninfected and infected mouse-decidual macrophages. The flow cytometric FMO as the control. **(F)** Histogram analysis of flow cytometry for LILRB4 expression levels in uninfected and infected mouse-decidual macrophages. Representative data for *in vitro* analysis of one individual from the uninfected and the infected groups. Data are presented are means ± SD (*p < 0.05) of 10 pregnant mice and 9 human spontaneous abortion specimens. Differences were identified by unpaired *t*-tests.

By immunofluorescence (Figures 2A,B) and flow cytometry (Figures 2C,D), LILRB4 expression levels were significantly decreased on human decidual macrophages after *T. gondii* infection, in comparison with uninfected macrophages. As judged by flow cytometry, murine decidual macrophage levels of LILRB4 were downregulated in mice infected with *T. gondii* (Figures 2E,F).

### Expression of M1 and M2 Membrane Molecules Is Changed When LILRB4 Is Downregulated by *T. gondii* Infection of Decidual Macrophages

**Figure 3:**
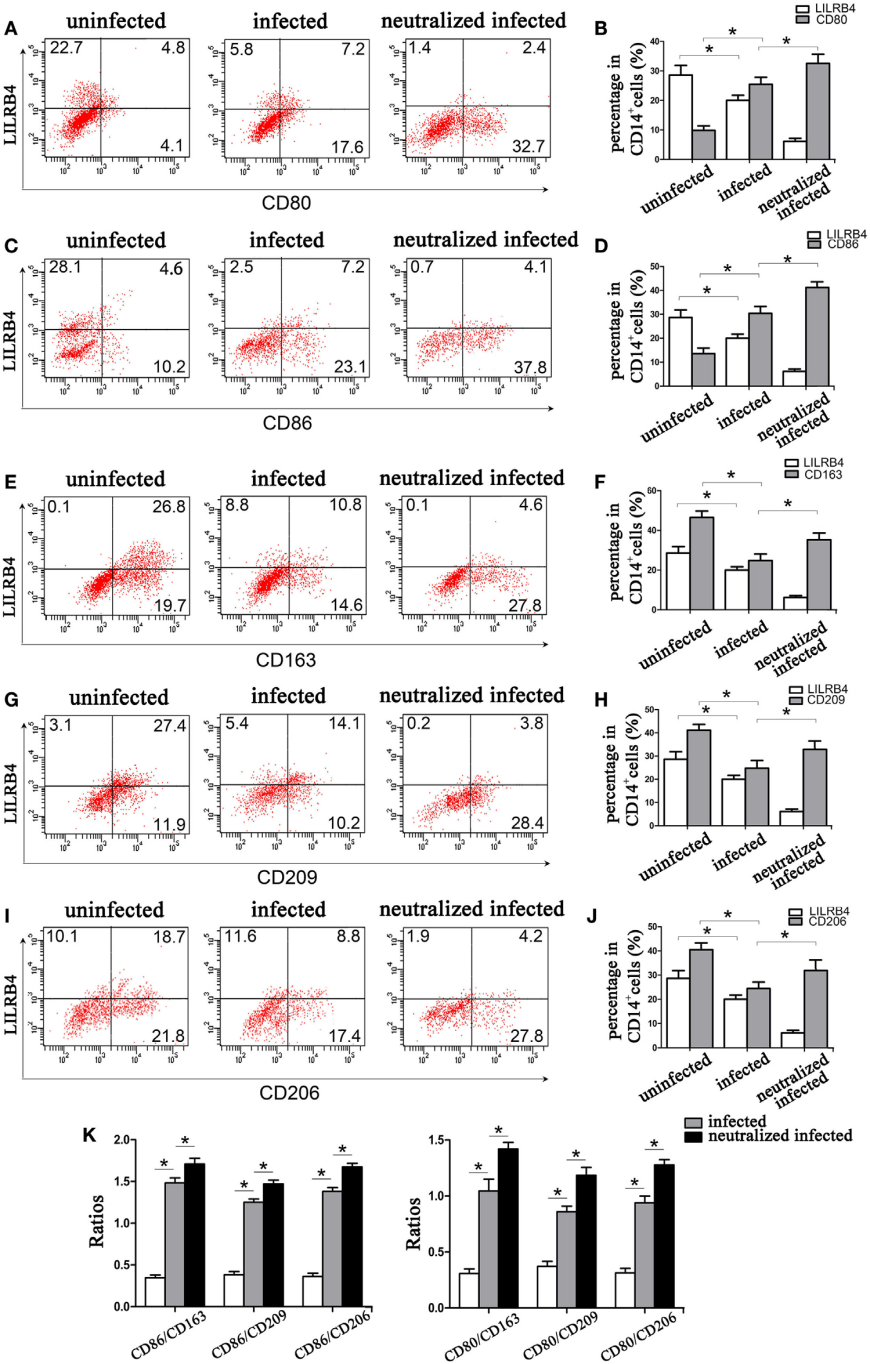
Downregulation of LILRB4 on human decidual macrophages infected with *Toxoplasma gondii*. Changes in the expression of M1 and M2 membrane-functional molecules. Histograms of flow cytometry in uninfected, infected, and LILRB4-neutralized and infected human decidual macrophage are presented. Representative histograms of M2 membrane-functional molecules: CD163 **(E,F)**, CD209 **(G,H)**, and CD206 **(I,J)**. Representative histograms of M1 membrane-functional molecules CD80 **(A,B)** and CD86 **(C,D)**. **(K)** M1/M2 ratios (CD86/CD163, CD86/CD209, CD86/CD206, CD80/CD163, CD80/CD209, and CD80/CD206) for uninfected, infected, and LILRB4-neutralized and infected human decidual macrophages. Representative data were from one individual for uninfected, infected and LILRB4-neutralized and infected groups, respectively. Data are presented as means ± SD (*p < 0.05, **p < 0.01); nine human spontaneous abortion specimens were assayed for each group and were compared by unpaired *t*-tests.

Flow cytometry demonstrated LILRB4 to be significantly downregulated on human decidual macrophages by *T. gondii* infection. M1 membrane-functional molecules CD80 (Figures 3A,B) and CD86 (Figures 3C,D) were significantly upregulated. In macrophages in which LILRB4 was neutralized, M1 membrane-functional molecules were further upregulated relative to infected cells (Figures 3A–D). M2 membrane-functional molecules CD163, CD209, and CD206 were significantly downregulated on human decidual macrophages after *T. gondii* infection and were slightly increased in the LILRB4-neutralized macrophages compared with infected macrophages (Figures 3E,F for CD163, Figures 3G,H for CD209, and Figures 3I,J for CD206). Furthermore, M1/M2 ratios (CD80/CD163, CD80/CD209, CD80/CD206, CD86/CD163, CD86/CD209, and CD86/CD206) were lowest in uninfected, greater in infected, and greatest in LILRB4-neutralized and infected macrophages (Figure 3K).

**Figure 4:**
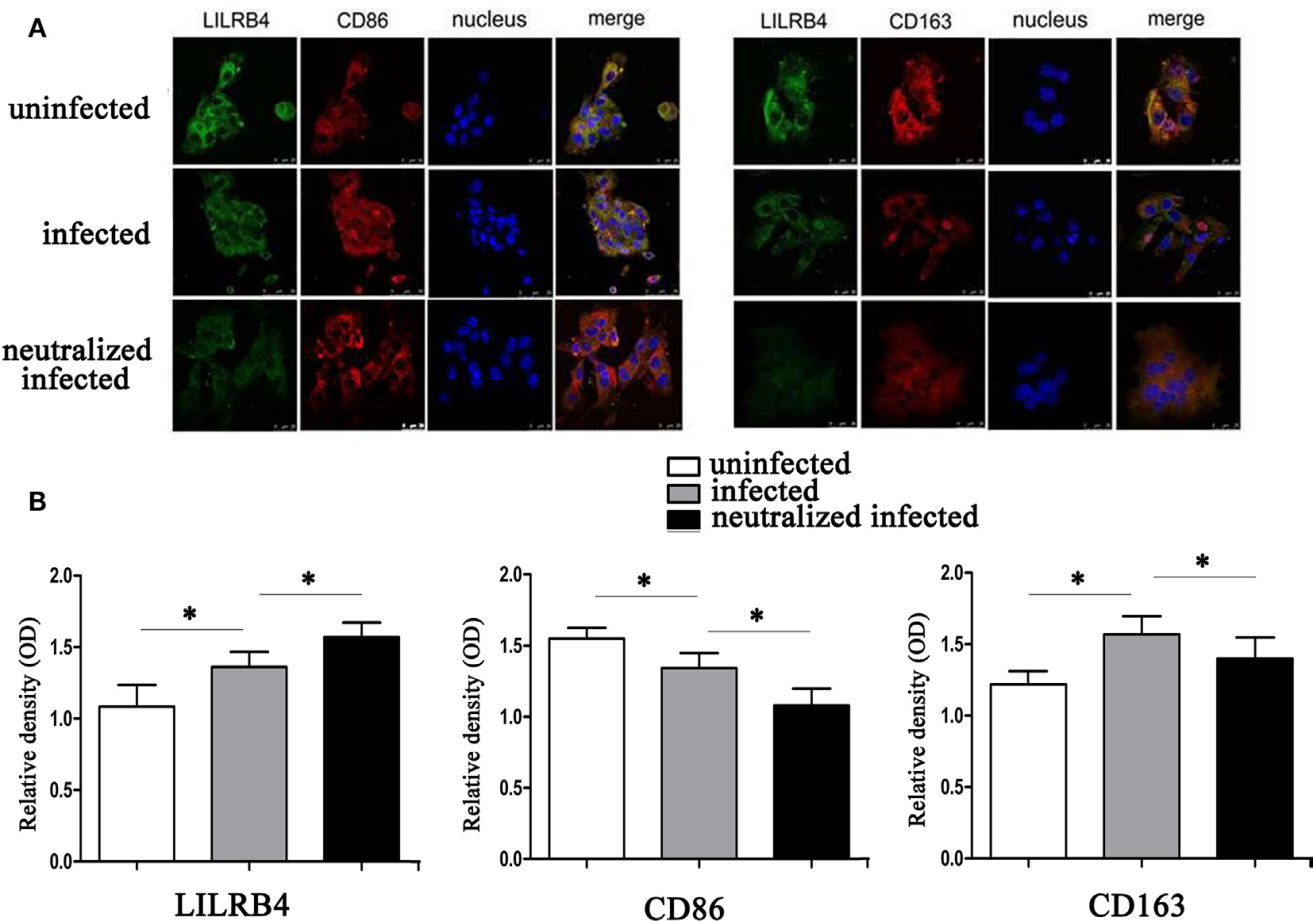
Downregulation of LILRB4 on human decidual macrophages infected with *Toxoplasma gondii*. Changes in the expression of M1 and M2 membrane-functional molecules CD163 and CD86. **(A)** Representative immunofluorescent photographs of LILRB4 (green), CD163 (red), and CD86 (red) expression in uninfected, infected, and LILRB4-neutralized and infected human decidual macrophages. Nuclei (blue) were stained with 4′,6-diamidino-2-phenylindole. **(B)** Histograms analysis of LILRB4, CD163, and CD86 expression in uninfected, infected, and LILRB4-neutralized and infected human decidual macrophages. Representative data are for one individual from uninfected, infected, and infected LILRB4-neutralized groups, respectively. Data are presented as means ± SD (*p < 0.05, **p < 0.01). Comparisons were by unpaired *t*-test; seven human spontaneous abortion specimens were assayed individually for each group.

**Figure 5:**
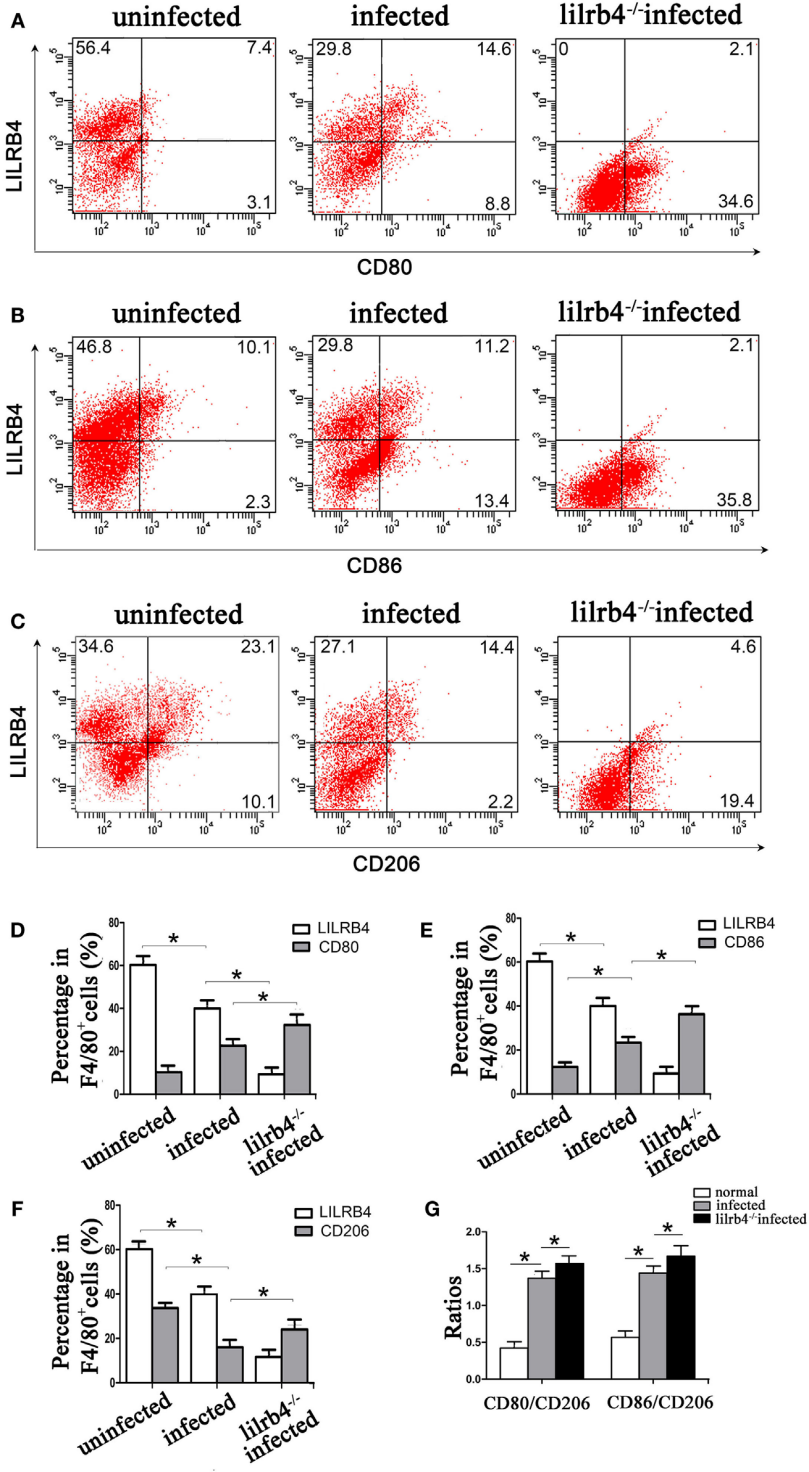
Downregulation of LILRB4 in mouse-decidual macrophages by *Toxoplasma gondii* infection changed the expression of M1 and M2 membrane-functional molecules. Representative histograms of M2 membrane-functional molecules CD206 **(C,F)** and M1 membrane-functional molecules CD80 **(A,D)** and CD86 **(B,E)** for uninfected, infected, and LILRB4^−/−^ infected mouse-decidual macrophage by flow cytometry. **(G)** Ratios of CD86/CD206 and CD80/CD206 in decidual macrophages from uninfected, infected, and LILRB4^−/−^ infected mice. Representative data were from different individuals in uninfected, infected, and LILRB4^−/−^ infected mice, respectively. Data are presented as means ± SD of 10 mice for each group, and differences were identified by unpaired *t*-test; *p < 0.05, **p < 0.01.

By immunofluorescence, infected macrophages had decreased LILRB4 and CD163 and increased CD86 expression when compared with uninfected macrophages. In the infected, LILRB4-neutralized macrophages, LILRB4 expression level was very low, whereas CD163 was slightly upregulated and CD86 was significantly upregulated in comparison with infected macrophages (Figures 4A,B). Similar to these *in vitro* studies, M1 membrane-functional molecules CD80 and CD86 were both significantly increased after *T. gondii* infection and increased further in LILRB4^−/−^ infected mice (Figures 5A,D for CD80, Figures 5B,E for CD86). In contrast, the M2 membrane-functional molecule CD206 was significantly decreased after *T. gondii* infection and was slightly increased in LILRB4^−/−^ infected mice when compared with infected mice (Figures 5C,F). CD80/CD206 and CD86/CD206 ratios were lowest in uninfected, greater in infected, and greatest in LILRB4^−/−^ infected mice (Figure 5G).

### LILRB4 Downregulation by *T. gondii* Infection of Decidual Macrophages Alters Expression of the Arginine Catabolism Enzymes Arg-I and iNOS

**Figure 6:**
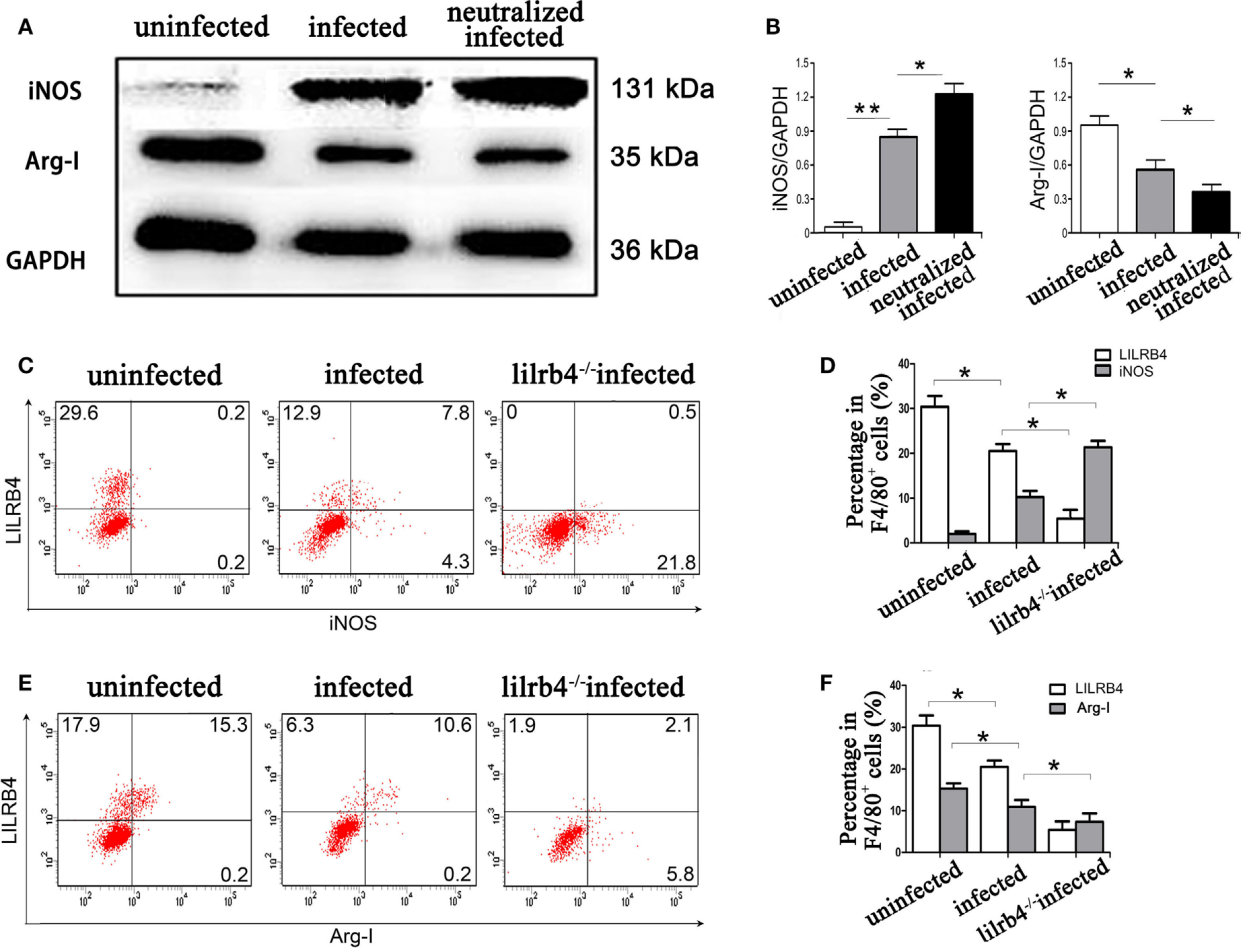
Downregulation of LILRB4 on decidual macrophages by *Toxoplasma gondii* infection changed the expression of the arginine catabolism enzymes type-I arginase (Arg-I) and inducible nitric oxide synthase (iNOS). **(A)** Representative depictions of iNOS (131 kDa) and Arg-I (35 kDa) protein levels in uninfected, infected, and LILRB4-neutralized and infected human decidual macrophages by western blot. **(B)** Histograms analysis of western blot for iNOS and Arg-I expression in uninfected, infected, and LILRB4-neutralized and infected human decidual macrophages. Representative histograms for iNOS **(C,D)** and Arg-I **(E,F)** protein levels in uninfected, infected, and LILRB4^−/−^ infected mouse-decidual macrophages by flow cytometry. Representative data for *in vivo* analysis of different individuals in uninfected, infected, and LILRB4^−/−^ infected groups. Representative data for *in vitro* analysis of one individual from uninfected, infected and LILRB4-neutralized and infected groups. Data are expressed as means ± SD of 10 pregnant mice or 9 human spontaneous abortion samples for each treatment group; *p < 0.05, **p < 0.01, by unpaired *t*-test.

By western blot analysis, the arginine catabolism enzyme iNOS was not detected in uninfected human decidual macrophages. After *T. gondii* infection the enzyme increased. The increase was even greater in infected LILRB4-neutralized macrophages. Arg-I synthesis in human decidual macrophages was significantly reduced following *T. gondii* infection and was further reduced in infected LILRB4-neutralized macrophages (Figures 6A,B). *In vivo* flow cytometric analysis demonstrated iNOS in decidual macrophages from *T. gondii* infected mice, with greater detection in decidual macrophages from infected LILRB4^−/−^ mice (Figures 6C,D). Arg-I expression was decreased in decidual macrophages from infected mice and further decreased in decidual macrophages from LILRB4^−/−^ infected mice (Figures 6E,F).

### Decidual Macrophage Secretion of TNF-α and IL-10 Is Affected When LILRB4 Is Downregulated by *T. gondii* Infection

**Figure 7:**
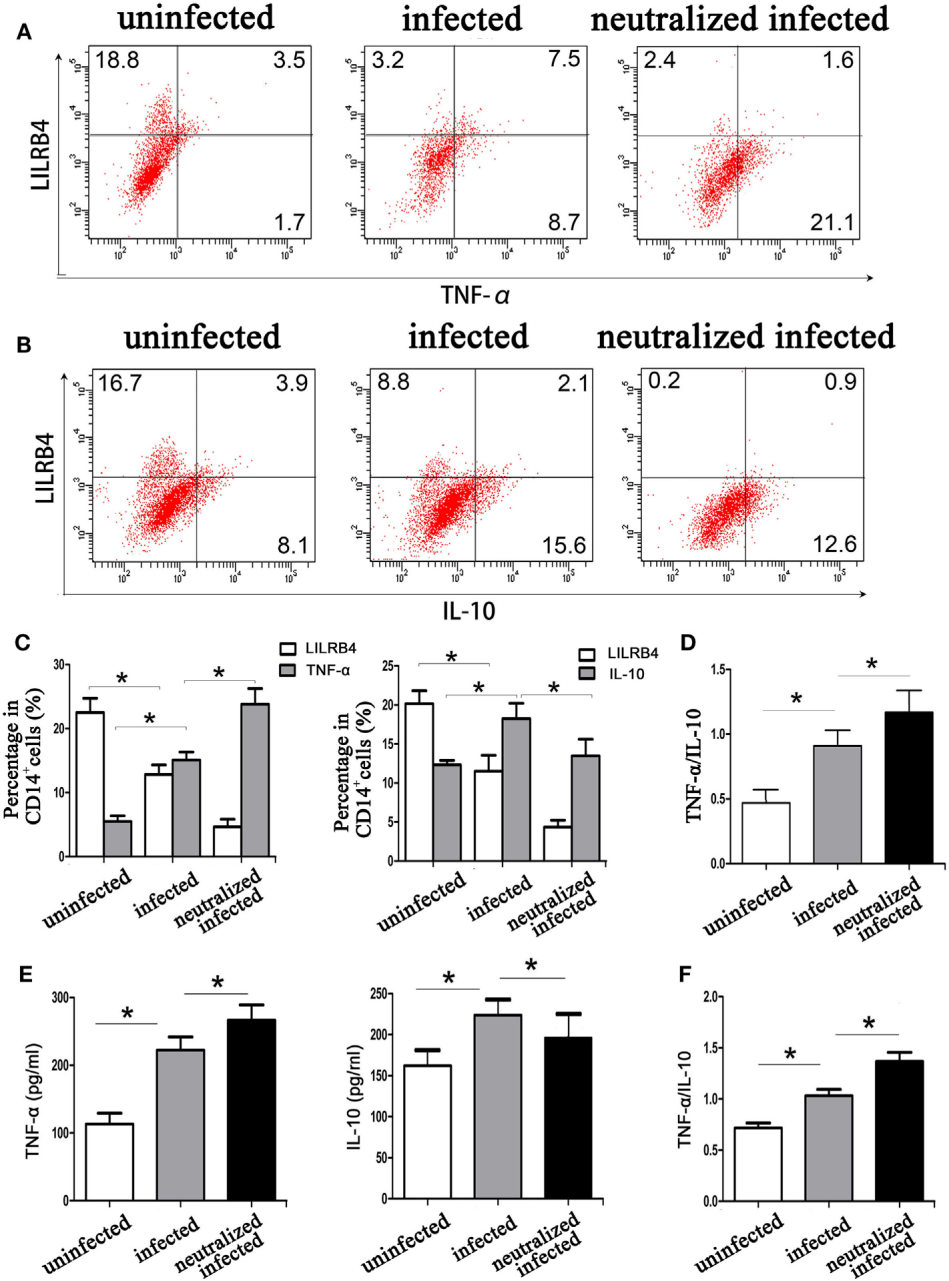
Downregulation of LILRB4 on decidual macrophages by *Toxoplasma gondii* infection affects tumor necrosis factor alpha (TNF-α) and interleukin 10 (IL-10) secretion by decidual macrophages. TNF-α **(A)** and IL-10 **(B)** levels in uninfected, infected, and LILRB4-neutralized and infected human decidual macrophages were assessed by flow cytometry analyses. **(C)** Histograms analysis for TNF-α and IL-10 in uninfected, infected, and LILRB4-neutralized infected human decidual macrophages **(D)** Ratios of TNF-α/IL-10 in uninfected, infected and LILRB4-neutralized, and infected human decidual macrophages. **(E)** IL-10 levels, TNF-α levels, and TNF-α/IL-10 ratios **(F)** were analyzed in supernatants from uninfected, infected, and LILRB4-neutralized and infected human decidual macrophages by enzyme-linked immunosorbent assays (ELISA). Representative data for *in vitro* analysis were from one individual in uninfected, infected, and LILRB4-neutralized and infected groups. ELISA samples were assayed in triplicate. Data are expressed as means ± SD of nine human spontaneous abortion specimens from each group; *p < 0.05 by unpaired *t*-test.

**Figure 8:**
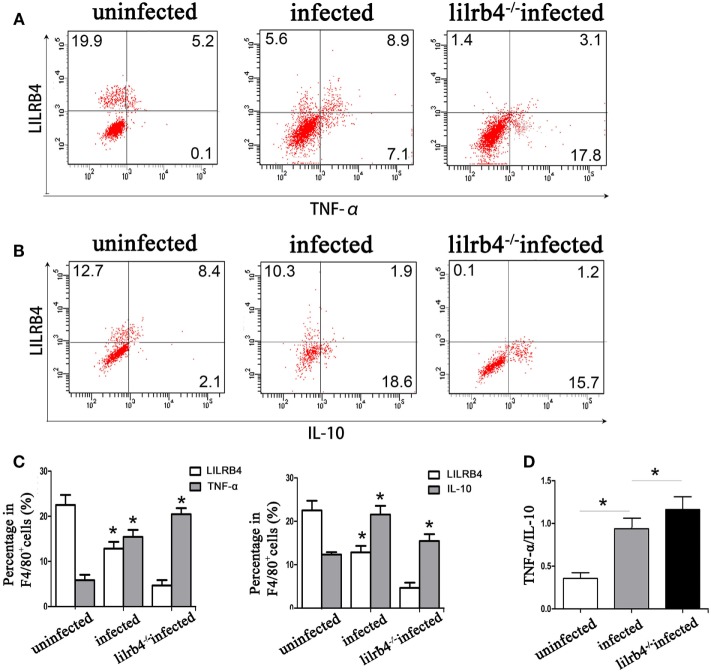
Downregulation of LILRB4 on mouse-decidual macrophages with *Toxoplasma gondii* infections changed IL-10 and TNF-α secretion. TNF-α **(A)** and IL-10 **(B)** in decidual macrophages from uninfected, infected, and LILRB4^−/−^ infected were assessed by flow cytometry. **(C)** Histogram analysis of flow cytometry for IL-10 and TNF-α in normal, infected, and LILRB4^−/−^ infected mouse-decidual macrophages **(D)** TNF-α/IL-10 ratios analyzed in uninfected, infected, and LILRB4^−/−^ infected mice decidual macrophages. Representative data for *in vivo* analysis of different individuals from uninfected, infected, and LILRB4^−/−^ infected groups. Data are expressed as means ± SD of 10 animals from each group; *p < 0.05 by unpaired *t*-test.

By flow cytometry, TNF-α levels within human decidual macrophages were increased following *T. gondii* infection and were further increased in infected LILRB4-neutralized cells (Figures 7A,C). IL-10 levels within human decidual macrophages were increased by *T. gondii* infection and reduced in infected LILRB4-neutralized cells (Figures 7B,C). TNF-α/IL-10 ratios were greater in infected cells than in uninfected cells and were further increased in infected LILRB4-neutralized cells (Figure 7D). ELISA analysis showed increased TNF-α secretion in supernatants from infected human decidual macrophages, which was further increased in supernatants from infected LILRB4-neutralized cells. IL-10 in supernatants from human decidual macrophages was increased after *T. gondii* infection and decreased in supernatants from infected human LILRB4-neutralized macrophages, when compared with the infected cells (Figure 7E). TNF-α/IL-10 ratios were increased in infected cells compared with uninfected cells and were further increased in infected LILRB4-neutralized cells (Figure 7F) By flow cytometry, TNF-α was increased in decidual macrophages from infected mice and was further increased in decidual macrophages from infected LILRB4^−/−^ mice (Figures 8A,C). IL-10 secretion by mouse-decidual macrophages was increased by *T. gondii* infection, while IL-10 secretion was decreased in decidual macrophages from infected LILRB4^−/−^ mice (Figures 8B,C). Finally, TNF-α/IL-10 ratios were increased in infected decidual macrophages compared with uninfected cells. A further increase was observed in decidual macrophages from infected LILRB4^−/−^ mice (Figure 8D).

## Discussion

*Toxoplasma gondii* is a zoonotic parasite that causes toxoplasmosis. The greatest *T. gondii* hazard is vertical transmission during pregnancy (1, 19), with increased incidence of abortion, stillbirth, and congenital anencephaly (3, 20, 21). Successful pregnancy requires subtle regulation by immune cell populations and their secreted cytokines at the maternal–fetal interface (22). Studies have confirmed that during gestation, *T. gondii* disturbs the maternal–fetal interface, resulting in abnormal pregnancy outcomes (3, 21). Our previous work showed that functional molecules and cytokines produced by maternal NK, Treg, and DC cells are affected by *T. gondii* infection and that these cells contribute to abnormal pregnancy (21, 23). Decidual macrophages are a major subset of decidual immune cells and contribute to local immune tolerance during normal pregnancies (24, 25). LILRB4 is predominantly expressed on macrophages and is an inhibitory receptor that is central to immune tolerance (26). Specifically, LILRB4 acts as an endogenous negative regulator of macrophage activation, modulating cytokine secretion during pregnancy (15–17). However, it is unknown whether decidual macrophage LILRB4 participates in abnormal pregnancy outcomes during *T. gondii* infection. In this study, we observed high levels of LILRB4 on uninfected mouse-decidual macrophages but low levels of LILRB4 in association with abnormal pregnancy outcomes mediated by *T. gondii* infection. To evaluate the relationship of LILRB4 to abnormal pregnancy outcomes, we established LILRB4^−/−^ and *T. gondii* infected pregnant mouse models and then compared pregnancy outcomes with those of *T. gondii* infected wild-type pregnant mice. Interestingly, pregnancy outcomes in infected LILRB4^−/−^ pregnant mice were more severe and showed more stillbirths and absorbed sites, smaller placental and fetal size, and more severe hyperemia, when compared with wild-type infected mice. Compared with uninfected mice, *T. gondii* infected mice had a significant increase in decidual macrophages suggesting that infection induced a pro-inflammatory response. A modest increase in decidual macrophages between infected and LILRB4^−/−^ infected mice was not significant. These results suggest that LILRB4 plays a role during pregnancy, and that the role of LILRB4 is demonstrable during *T. gondii* infection.

LILRB4 expression was monitored on decidual macrophages during *T. gondii* infection both *in vitro* and *in vivo*. Those experiments showed LILRB4 expression to be significantly downregulated by *T. gondii* infection in both human and mouse-decidual macrophages during pregnancy. The prevalence of abnormal pregnancy outcomes among infected LILRB4^−/−^ mice further suggests that LILRB4 downregulation on decidual macrophages may play a role in the development of *T. gondii*-mediated abnormalities. To assess a mechanistic basis for these observations, we determined expression levels of functional molecules in decidual macrophages including membrane molecules, arginine metabolic enzymes, and cytokines, during infection.

Decidual macrophages are classified as M1 or M2 subsets (9, 10). M1 decidual macrophages are characterized by high expression of CD80 and CD86 and are associated with inflammatory responses and poor maternal–fetal tolerance (8). Excessive numbers of M1 decidual macrophages promote trophoblast apoptosis and retard placental development (12). In addition, upregulation of the costimulatory molecules CD80 and CD86 reportedly contributes to an abnormal immune microenvironment and a shift to a Th1 response at the maternal–fetal interface, resulting in human miscarriage (27). In contrast, M2 decidual macrophages express CD206, CD209, and CD163, and these macrophages mediate immunosuppression and maternal–fetal tolerance (25, 28). M2 decidual macrophage CD206 sustains immune homeostasis and immunosuppression at the maternal–fetal interface (29). Similarly, CD209, mainly expressed on M2 decidual macrophages, contributes to maternal–fetal immune tolerance (30). CD163 is known as a homeostatic scavenger receptor and has been associated with tissue homeostasis and immune regulation (11, 25). *T. gondii* infection reportedly affects the polarization of macrophages during rat pregnancy, which further suggests that macrophage polarization is involved in abnormal pregnancy outcomes (31). Herein, we examined the effects of *T. gondii* on expression of M1 and M2 related membrane-functional molecules during pregnancy as well as in cultured human and mouse-decidual macrophages. Those experiments showed that with LILRB4 downregulation, the M1 membrane-functional molecules CD80 and CD86 were significantly upregulated, whereas the M2 membrane-functional molecules CD206, CD209, and CD163 were significantly downregulated both *in vivo* and *in vitro*. These data suggest that in addition to downregulating LILRB4, *T. gondii* infection contributes to the development of abnormal pregnancy outcomes by skewing decidual macrophages from an M2 type to an inflammatory M1 type. To clarify the role of LILRB4, we performed experiments in which LILRB4 was neutralized with antibody *in vitro* and *in vivo* by use of LILRB4^−/−^ mice during *T. gondii* infection.

The M1 membrane-functional molecules CD80 and CD86 were upregulated in infected LILRB4-neutralized human decidual macrophages and in decidual macrophages from infected LILRB4^−/−^ mice. M2 membrane-functional molecules CD206, CD209, and CD163 were moderately upregulated as judged by flow cytometry. Immunofluorescence analysis of CD163, CD86, and LILRB4 on human decidual macrophages was consistent with the flow cytometric analysis. M1/M2 ratios for CD80/CD163, CD80/CD206, CD80/CD209, CD86/CD163, CD86/CD206, and CD86/CD209 suggested a tendency toward M1 functional molecules in LILRB4-neutralized and infected human decidual macrophages and in infected LILRB4^−/−^ mice. Downregulation of LILRB4 on decidual macrophages during *T. gondii* infection likely shifts macrophages toward an M1 type and away from an M2 type, which likely contributes to abnormal pregnancy outcomes during *T. gondii* infection.

Decidual macrophages synthesize the arginine metabolic enzymes Arg-I and iNOS, which are associated with M1 and M2 phenotypes, respectively, and are widely used to distinguish between M1 and M2 macrophages (32, 33). Arg-I enhances the bioavailability of l-arginine, which is involved in immunosuppression and reportedly promotes polyamine synthesis to enhance placental growth and development (34, 35). iNOS in human placentas is absent under normal conditions, whereas excessive expression of iNOS suppresses placental vascular development (36, 37). In this study, results show induced expression of iNOS in M1 human and mouse-decidual macrophages after *T. gondii* infection, with no expression in uninfected cells. In contrast, Arg-I synthesis was downregulated in *T. gondii*-infected decidual macrophages *in vivo* and *in vitro*. Hence, along with LILRB4 downregulation, *T. gondii*-induced iNOS expression enhanced M1 decidual macrophage activation and downregulated Arg-I expression hampering immune suppression by M2 decidual macrophages. An anti-LILRB4 neutralizing antibody and LILRB4^−/−^ mice, infected with *T. gondii*, were used to determine whether changes in the arginine metabolic enzymes Arg-I and iNOS during *T. gondii* infection were a consequence of decreased LILRB4 expression. In those experiments, iNOS upregulation was observed in anti-LILRB4-neutralized and infected human decidual macrophages and in LILRB4^−/−^ infected mice. In contrast, Arg-I expression was downregulated under these conditions. Hence, changes in Arg-I and iNOS expression during *T. gondii* infection are associated with decreased LILRB4 expression in decidual macrophages, which appears to relate to the prevalence of M1 decidual macrophages. An imbalance of M1 and M2 decidual macrophages, due to change in arginine metabolic enzymes, may contribute to the development of abnormal pregnancy outcomes during *T. gondii* infection.

Previously, IL-10 in decidual macrophage has been linked to the development of Th2-polarized local immunity. T-cell anergy to fetal antigens has been associated with the IL-10 regulatory and tolerant properties of decidual macrophages (29). IL-10 therapy of *T. gondii* infections reduces trophoblasts apoptosis at the maternal–fetal interface and improves pregnancy outcomes (38, 39). Decidual macrophage LILRB4 ligation by FcγRI inhibited TNF-α production and hampered functional activation (17). Moreover, activated decidual macrophages can produce high levels of TNF-α at the maternal–fetal interface, and interactions with TNF-α receptors on extravillous trophoblasts induce trophoblast apoptosis *in vitro* (40, 41). The major cytokines TNF-α and IL-10 are secreted by decidual macrophages at the maternal–fetal interface and are involved in the balance of M1 and M2 phenotypes (11, 42). Herein, both TNF-α and IL-10 levels were increased in decidual macrophages. TNF-α/IL-10 ratios were increased after *T. gondii* infection of both human and mouse-decidual macrophages, suggesting a pro-inflammatory rather than an immunosuppressive environment. These data were confirmed by ELISA and by flow cytometry.

Finally, we determined whether changes in TNF-α and IL-10 secretion during *T. gondii* infection were due to a decrease in expression of LILRB4 by decidual macrophages. Both flow cytometry and ELISA analysis of supernatants showed increased TNF-α and decreased IL-10 expression and secretion levels in infected LILRB4^−/−^ mice decidual macrophages and in infected human decidual macrophages that were treated with anti-LILRB4 neutralizing antibody. These data indicate that *T. gondii*-mediated reductions in LILRB4 result in the observed changes in TNF-α and IL-10 expression and suggest a prominent role for LILRB4 at the maternal–fetal interface. An imbalance in cytokines at the maternal–fetal interface likely contributes to the development of abnormal pregnancy outcomes during *T. gondii* infection.

Taken together, the results of this study show that downregulation of the inhibitory receptor LILRB4 on decidual macrophages during *T. gondii* infection strengthens an M1 activating and weakens an M2 tolerance response by altering M1 and M2 membrane molecule expression, the synthesis of arginine metabolic enzymes, and the cytokine secretory profile. These alterations result in abnormal pregnancy outcomes. This investigation contributes to an understanding of the immune mechanisms that result in abnormal pregnancy outcomes due to *T. gondii* infection.

## Ethics Statement

Sample collection procedures for this study were approved by the Binzhou Medical University Ethics Committee (Shandong, China). All subjects provided written informed consent for the collection of samples and subsequent analysis. The Ethics Committees approved this consent procedure. This study was carried out in strict accordance with the recommendations in the Guide for the Care and Use of Laboratory Animals of Binzhou Medical University. The protocol was approved by the Committee on the Ethics of Animal Experiments of Binzhou Medical University. All procedures were performed under sodium pentobarbital anesthesia, and all efforts were made to minimize suffering of the animals.

## Author Contributions

ZL, TL, and JZ collected samples. XH and ZL conceived and designed the experiments as well as wrote the paper. ZL, MZ, and TL conducted the experiments. XH, ZL, MZ, TL, JZ, XL, YJ, and HZ analyzed the data and contributed reagents/materials/analysis.

## Conflict of Interest Statement

The authors declare that the research was conducted in the absence of any commercial or financial relationships that could be construed as a potential conflict of interest.

## References

[1] Mahalakshmi B Therese KL Devipriya U Pushpalatha V Margarita S Madhava HN. Infectious aetiology of congenital cataract based on TORCHES screening in a tertiary eye hospital in Chennai, Tamil Nadu, India. Indian J Med Res (2010) 131:559–64. 20424308

[2] Li XL Wei HX Zhang H Peng HJ Lindsay DS. A meta analysis on risks of adverse pregnancy outcomes in *Toxoplasma gondii* infection. PLoS One (2014) 9(5):e97775. 10.1371/journal.pone.0097775 24830795 PMC4022675

[3] Jones JL Lopez A Wilson M Schulkin J Gibbs R. Congenital toxoplasmosis: a review. Obstet Gynecol Surv (2001) 56:296–305. 10.1097/00006254-200105000-00025 11333376

[4] Mor G Cardenas I Abrahams V Guller S. Inflammation and pregnancy: the role of the immune system at the implantation site. Ann N Y Acad Sci (2011) 1221:80–7. 10.1111/j.1749-6632.2010.05938.x 21401634 PMC3078586

[5] Faas MM de Vos P. Uterine NK cells and macrophages in pregnancy. Placenta (2017) 56:44–52. 10.1016/j.placenta.2017.03.001 28284455

[6] Mori M Bogdan A Balassa T Csabai T Szekeres-Bartho J The decidual-the maternal bed embracing the embryo-maintains the pregnancy. Semin Immunopathol (2016) 38(6):635–49. 10.1007/s00281-016-0574-0 27287066 PMC5065593

[7] Trundley A Gardner L Northfield J Chang C Moffett A. Methods for isolation of cells from the human fetal-maternal interface. Methods Mol Med (2006) 122:109–22. 10.1385/1-59259-989-3:109 16511978

[8] Ning F Liu H Lash GE. The role of decidual macrophages during normal and pathological pregnancy. Am J Reprod Immunol (2016) 75(3):298–309. 10.1111/aji.12477 26750089

[9] Gordon S Alternative activation of macrophages. Nat Rev Immunol (2003) 3:23–35. 10.1038/nri978 12511873

[10] Goerdt S Orfanos CE Other functions, other genes: alternative activation of antigen-presenting cells. Immunity (1999) 10:137–42. 10.1016/S1074-7613(00)80014-X 10072066

[11] Svensson J Jenmalm MC Matussek A Geffers R Berg G Ernerudh J. Macrophages at the fetal-maternal interface express markers of alternative activation and are induced by M-CSF and IL-10. J Immunol (2011) 187(7):3671–82. 10.4049/jimmunol.1100130 21890660

[12] Nagamatsu T Schust DJ. The contribution of macrophages to normal and pathological pregnancies. Am J Reprod Immunol (2010) 63(6):460–71. 10.1111/j.1600-0897.2010.00813.x 20163399

[13] Cella M Döhring C Samaridis J Dessing M Brockhaus M Lanzavecchia A A novel inhibitory receptor (ILT3) expressed on monocytes, macrophages, and dendritic cells involved in antigen processing. J Exp Med (1997) 185(10):1743–51. 10.1084/jem.185.10.1743 9151699 PMC2196312

[14] Castells MC Wu X Arm JP Austen KF Katz HR. Cloning of the gp49B gene of the immunoglobulin superfamily and demonstration that one of its two products is an early-expressed mast cell surface protein originally described as gp49. J Biol Chem (1994) 269(11):8393–401. 8132564

[15] Ban YL Kong BH Qu X Yang QF Ma YY. BDCA-1+, BDCA-2+ and BDCA-3+ dendritic cells in early human pregnancy decidua. Clin Exp Immunol (2008) 151(3):399–406. 10.1111/j.1365-2249.2007.03576.x 18234052 PMC2276959

[16] Rochat MK Ege MJ Plabst D Steinle J Bitter S Braun-Fahrländer C Maternal vitamin D intake during pregnancy increases gene expression of ILT3 and ILT4 in cord blood. Clin Exp Allergy (2010) 40(5):786–94. 10.1111/j.1365-2222.2009.03428.x 20030662

[17] Matsumoto Y Wang LL Yokoyama WM Aso T. Uterine macrophages express the gp49B inhibitory receptor in midgestation. J Immunol (2001) 166:781–6. 10.4049/jimmunol.166.2.781 11145650

[18] Rojo S Stebbins CC Peterson ME Dombrowicz D Wagtmann N Long EO. Natural killer cells and mast cells from gp49B null mutant mice are functional. Mol Cell Biol (2000) 20(19):7178–82. 10.1128/MCB.20.19.7178-7182.2000 10982834 PMC86271

[19] Gontijo da Silva M Clare Vinaud M de Castro AM. Prevalence of toxoplasmosis in pregnant women and vertical transmission of *Toxoplasma gondii* in patients from basic units of health from Gurupi, Tocantins, Brazil, from 2012 to 2014. PLoS One (2015) 10(11):e0141700. 10.1371/journal.pone.0141700 26558622 PMC4641701

[20] Remington JS McLeod R Desmonts G Toxoplasmosis. 4th ed In: Remington JS Klein JO, editors. Infectious Diseases of the Fetus and Newborn Infant. Philadelphia, PA: The W. B. Saunders Company (1995). p. 140–267.

[21] Liu X Zhao M Yang X Han M Xu X Jiang Y *Toxoplasma gondii* infection of decidual CD1c(+) dendritic cells enhances cytotoxicity of decidual natural killer cells. Inflammation (2014) 37(4):1261–70. 10.1007/s10753-014-9853-x 24573986

[22] Luppi P. How immune mechanisms are affected by pregnancy. Vaccine (2003) 21(24):3352–7. 10.1016/S0264-410X(03)00331-1 12850338

[23] Liu Y Zhao M Xu X Liu X Zhang H Jiang Y Adoptive transfer of Treg cells counters adverse effects of *Toxoplasma gondii* infection on pregnancy. J Infect Dis (2014) 210(9):1435–43. 10.1093/infdis/jiu265 24799601

[24] Gustafsson C Mjösberg J Matussek A Geffers R Matthiesen L Berg G Gene expression profiling of human decidual macrophages: evidence for immunosuppressive phenotype. PLoS One (2008) 3(4):e2078. 10.1371/journal.pone.0002078 18446208 PMC2323105

[25] Svensson-Arvelund J Ernerudh J. The role of macrophages in promoting and maintaining homeostasis at the fetal-maternal interface. Am J Reprod Immunol (2015) 74(2):100–9. 10.1111/aji.12357 25582625

[26] Svensson-Arvelund J Mehta RB Lindau R Mirrasekhian E Rodriguez-Martinez H Berg G The human fetal placenta promotes tolerance against the semiallogeneic fetus by inducing regulatory T cells and homeostatic M2 macrophages. J Immunol (2015) 194(4):1534–44. 10.4049/jimmunol.1401536 25560409

[27] Chang CC Ciubotariu R Manavalan JS Yuan J Colovai AI Piazza F Tolerization of dendritic cells by T(S) cells: the crucial role of inhibitory receptors ILT3 and ILT4. Nat Immunol (2002) 3(3):237–43. 10.1038/ni760 11875462

[28] Jin LP Fan DX Zhang T Guo PF Li DJ. The costimulatory signal upregulation is associated with Th1 bias at the maternal-fetal interface in human miscarriage. Am J Reprod Immunol (2011) 66(4):270–8. 10.1111/j.1600-0897.2011.00997.x 21481059

[29] Nagamatsu T Schust DJ The immunomodulatory roles of macrophages at the maternal-fetal interface. Reprod Sci (2010) 17(3):209–18. 10.1177/1933719109349962 20065301

[30] Abrahams VM Kim YM Straszewski SL Romero R Mor G. Macrophages and apoptotic cell clearance during pregnancy. Am J Reprod Immunol (2004) 51(4):275–82. 10.1111/j.1600-0897.2004.00156.x 15212680

[31] Breburda EE Dambaeva SV Slukvin II Golos TG Selective distribution and pregnancy-specific expression of DC-SIGN at the maternal-fetal interface in the rhesus macaque: DC-SIGN is a putative marker of the recognition of pregnancy. Placenta (2006) 27(1):11–21. 10.1016/j.placenta.2004.11.006 16310033

[32] Kong L Zhang Q Chao J Wen H Zhang Y Chen H Polarization of macrophages induced by *Toxoplasma gondii* and its impact on abnormal pregnancy in rats. Acta Trop (2015) 143:1–7. 10.1016/j.actatropica.2014.12.001 25496968

[33] Hibbs JB Jr Vavrin Z Taintor RR. l-arginine is required for expression of the activated macrophage effector mechanism causing selective metabolic inhibition in target cells. J Immunol (1987) 138(2):550–65. 2432129

[34] Yang Z Ming X. Functions of arginase isoforms in macrophage inflammatory responses: impact on cardiovascular diseases and metabolic disorders. Front Immunol (2014) 5:533. 10.3389/fimmu.2014.00533 25386179 PMC4209887

[35] Neri I Mazza V Galassi MC Volpe A Facchinetti F. Effects of l-arginine on utero-placental circulation in growth-retarded fetuses. Acta Obstet Gynecol Scand (1996) 75:208–12. 10.3109/00016349609047088 8607330

[36] Wink DA Hines HB Cheng RY Switzer CH Flores-Santana W Vitek MP Nitric oxide and redox mechanisms in the immune response. J Leukoc Biol (2011) 89(6):873–91. 10.1189/jlb.1010550 21233414 PMC3100761

[37] Garvey EP Tuttle JV Covington K Merrill BM Wood ER Baylis SA Purification and characterization of the constitutive nitric oxide synthase from human placenta. Arch Biochem Biophys (1994) 311(2):235–41. 10.1006/abbi.1994.1232 7515611

[38] Wang X Wang J Trudinger B. Gene expression of nitric oxide synthase by human umbilical vein endothelial cells: the effect of fetal plasma from pregnancy with umbilical placental vascular disease. BJOG (2003) 110(1):53–8. 10.1046/j.1471-0528.2003.01329.x 12504936

[39] Zhao M Zhang R Xu X Liu Y Zhang H Zhai X IL-10 reduces levels of apoptosis in *Toxoplasma gondii* infected trophoblasts. PLoS One (2013) 8(2):e56455 10.1371/journal.pone.0056455 23418570 PMC3572055

[40] Zhang R Zhang H Liu X Fu Q Xu X Hu X. The immunoprotective role of interleukin-10 in abnormal pregnancy outcome induced by *Toxoplasma gondii* infection. Gynecol Obstet Invest (2012) 73(3):223–9. 10.1159/000333310 22156631

[41] Hunt JS. Macrophages in human uteroplacental tissues: a review. Am J Reprod Immunol (1989) 21:119–22. 10.1111/j.1600-0897.1989.tb01015.x 2701164

[42] Yui J Hemmings D Garcia-Lloret M Guilbert LJ. Expression of the human p55 and p75 tumor necrosis factor receptors in primary villous trophoblasts and their role in cytotoxic signal transduction. Biol Reprod (1996) 55:400–9. 10.1095/biolreprod55.2.400 8828846

